# Integrating CT radiomics and transcriptomics: a biologically-informed machine learning model for predicting chemotherapy response in advanced laryngeal cancer

**DOI:** 10.3389/fonc.2026.1740896

**Published:** 2026-04-15

**Authors:** Xin Xiong, Xiaodong Ji, Xilong Yang, Wei Wang, Xianfeng Wei

**Affiliations:** 1 Department of Otorhinolaryngology Head and Neck Surgery, Tianjin First Central Hospital, Institute of Otolaryngology of Tianjin, Key Laboratory of Auditory Speech and Balance Medicine, Key Medical Cultivation Discipline of Tianjin (Otolaryngology), Quality Control Centre of Otolaryngology, Tianjin, China; 2 Department of Radiology, Tianjin First Central Hospital, Nankai University, Tianjin, China; 3 Department of Radiology, First Central Clinical College, Tianjin Medical University, Tianjin, China

**Keywords:** chemotherapy response, laryngeal cancer, machine learning, radiomics, transcriptomics

## Abstract

**Background:**

Predicting response to induction chemotherapy (IC) in advanced laryngeal cancer (LC) remains a clinical challenge. This study aimed to develop a non-invasive, interpretable model integrating CT radiomics and clinical features to predict chemotherapy outcomes.

**Methods:**

We retrospectively analyzed 161 advanced LC patients treated with IC. From pre-treatment CT images, 1,321 radiomics features were extracted, and a radiomics score (Rad-score) was constructed using LASSO regression. Transcriptomic analysis explored the biological basis of Rad-score. Independent predictors were identified via multivariate logistic regression and used to build five machine learning models. Model performance was evaluated using AUC, accuracy, and specificity. SHAP analysis was applied to interpret the optimal model.

**Results:**

Four robust radiomics features were selected to construct the Rad-score. The Rad-score demonstrated satisfactory discrimination with an Area Under the Curve (AUC) of 0.715 in the training set and 0.707 in the validation set. In multivariate analysis, the Rad-score (Odds Ratio [OR]=2.89, 95% CI: 1.29–6.48, *P* = 0.010), gap invasion and validation were identified as independent predictors of chemotherapy response. Among the machine learning models, the Random Forest model achieved the best performance, yielding an AUC of 0.914 in the training set, 0.856 in the validation set, and 0.810 in the external test set. Decision curve analysis confirmed the clinical utility of the model. SHAP analysis confirmed Rad-score and fat space invasion as core predictors, with synergistic effects.

**Conclusions:**

We developed a highly accurate and interpretable Random Forest model that integrates radiomics and clinical features to predict IC response in advanced LC. This tool enables precise risk stratification and personalized treatment decisions, sparing non-responders from ineffective therapy. Prospective studies are needed to validate its clinical utility.

## Introduction

Laryngeal cancer (LC), particularly in its advanced stages, poses a significant challenge in clinical management due to its aggressive nature and limited therapeutic options. As a major subtype of head and neck squamous cell carcinoma (HNSCC), advanced LC is associated with a poor prognosis, with 5-year overall survival rates typically hovering between 50% and 60%, and this figure is even lower for patients with clinical stage IV disease (1, 2). Chemotherapy, especially induction chemotherapy (IC) administered before surgery or radiotherapy, remains a cornerstone of the comprehensive treatment strategy for advanced LC (3). Its primary objectives are to reduce tumor burden, potentially improve survival, and, importantly, facilitate laryngeal preservation, addressing the critical need to maintain organ function and quality of life (4). However, the clinical efficacy of chemotherapy varies significantly among patients. Unfortunately, some individuals resistant to IC experience toxic side effects without any therapeutic benefit. Therefore, there is an urgent need to develop reliable predictive tools to guide personalized treatment strategies and optimize clinical outcomes (5).

Computed Tomography (CT) imaging, as a highly valuable non-invasive modality, is widely used for assessing tumor characteristics and treatment response (6). Its ability to provide detailed anatomical and functional information makes it a promising tool for predicting chemotherapeutic efficacy (7). Recent studies have highlighted the utility of CT-derived parameters, such as tumor volume, texture features, and perfusion characteristics, in evaluating treatment response across various cancers (8, 9). In the context of laryngeal cancer, CT imaging can offer crucial insights into tumor heterogeneity and biological behavior, which are closely linked to chemosensitivity (10). Nevertheless, a significant clinical challenge remains in identifying reliable pre-treatment indicators of IC response, as robust models that integrate clinicopathological and peripheral blood parameters to predict IC response and overall survival in advanced laryngeal squamous cell carcinoma (LSCC) are still lacking (11). Despite its inherent potential, the application of pre-treatment CT imaging for accurately predicting chemotherapeutic efficacy in advanced laryngeal cancer remains underexplored (12). The existing literature has largely focused on early-stage disease or post-chemotherapy treatment assessment, leaving a significant gap in understanding how specific pre-treatment CT features can reliably guide initial therapeutic decisions (13, 14). Filling this gap is crucial, as accurate prediction of treatment response would enable clinicians to identify patients most likely to benefit from systemic therapy, thereby sparing others from unnecessary toxicities and allowing for more timely alternative interventions, such as surgery or radiotherapy (15).

This study aims to systematically investigate the role of pre-treatment CT imaging in predicting the response to chemotherapy in patients with advanced laryngeal cancer. By analyzing CT-derived imaging parameters in depth and exploring their correlation with treatment outcomes, we seek to establish a robust predictive framework to enhance personalized treatment planning. Our findings may contribute to the growing body of evidence supporting the integration of advanced imaging techniques into oncological practice, ultimately aiming to improve risk stratification, treatment selection, and overall prognosis for patients.

## Materials and methods

### Patient enrollment and data collection

This study is a retrospective, single-center cohort analysis of patients with pathologically confirmed laryngeal squamous cell carcinoma at Tianjin First Central Hospital from 2018 to 2023. A total of 161 patients were enrolled. The internal cohort consisted of 131 patients treated at Tianjin First Central Hospital from 2018 to 2023. To validate the model’s generalizability, an independent external testing cohort of 30 patients was enrolled from a separate patient population at our center between September and December 2023 (**Supplementary Table S1**). All patients received neoadjuvant chemotherapy based on a modified TPF regimen (docetaxel, nedaplatin, and fluorouracil), followed by definitive surgery or radiotherapy. The study strictly adhered to the principles of the Declaration of Helsinki and was approved by the Ethics Committee of Tianjin First Central Hospital. Written informed consent was waived due to the retrospective nature of the study.

Inclusion criteria required patients to be hospitalized for a laryngeal tumor, have a pathologically confirmed diagnosis of laryngeal cancer, and not have received any prior systemic treatment at the time of initial diagnosis. Additionally, eligible patients had no contraindications to chemotherapy, consented to receive neoadjuvant treatment, and underwent both plain and contrast-enhanced neck CT scans before the start of neoadjuvant chemotherapy, followed by a repeat scan within one week after completing the second chemotherapy cycle. Exclusion criteria included poor-quality imaging due to artifacts or other factors, failure to complete the planned neoadjuvant chemotherapy on schedule due to complications, contraindications, or adverse reactions, and receiving fewer than two cycles of neoadjuvant chemotherapy.

### Chemotherapy regimen and tolerance profile

All patients received a modified TPF induction chemotherapy regimen (16). The specific protocol was as follows: A total dose of 75 mg of docetaxel is administered intravenously over two days (Day 1 and Day 2). A total dose of 80–100 mg of nedaplatin is administered intravenously over two days (Day 1 and Day 2). In addition,5-Fluorouracil (750) mg/m²/day as a continuous intravenous infusion on days 3-7. The cycles were repeated every 3 weeks for a total of 2–3 cycles. Routine hydration, antiemetic, and prophylactic treatments were administered. All 161 patients completed at least two cycles of induction chemotherapy.

Chemotherapy toxicity was assessed according to the Common Terminology Criteria for Adverse Events (CTCAE) version 5.0. The modified TPF regimen was generally well-tolerated. The most frequently observed adverse events were leukopenia, which occurred in 35% of patients, with Grade 3–4 events accounting for 5%. Thrombocytopenia was observed in 30% of patients, with Grade 3–4 events occurring in 3%. Non-hematologic toxicities were primarily Grade 1-2, with nausea 40% of patients and fatigue 35%. No chemotherapy-related deaths occurred during the induction phase. These data indicate that the modified TPF regimen demonstrates an acceptable safety profile for patients with advanced laryngeal cancer.

### CT imaging and data collection

All patients received contrast-enhanced computed tomography (CECT) scans prior to initiating treatment, conducted within a seven-day timeframe. The imaging was performed using either a SOMATOM Definition or a SOMATOM Definition Flash, both 64-slice spiral CT scanners manufactured by SIEMENS. The imaging protocol included standardized parameters: a tube voltage of 120 kV, tube current ranging from 165 to 200 mAs, a field of view (FOV) set at 200 mm, and a reconstructed matrix size of 512 × 512. The axial slice thickness was maintained at 5 mm for all scans.

The imaging process began with non-contrast CT scans, followed by contrast-enhanced imaging. For the latter, an intravenous injection of a nonionic iodinated contrast agent (iohexol, 350 mg/dl iodine) was administered via the median vein of the right elbow using an automated injector. The contrast volume was calculated at 1 ml per kilogram of body weight, with a flow rate of 3 ml per second. The contrast-enhanced scans were initiated with a delay of 60 seconds after the injection. This protocol ensured consistent and high-quality imaging for all patients, facilitating accurate diagnostic and therapeutic planning.

### CT imaging protocol and data processing

All patients received non-contrast and contrast-enhanced CT scans of the neck at two key time points: before starting neoadjuvant immunotherapy (CT1) and within two weeks before surgery (CT2). Axial slice images were captured during the venous phase and accessed through the hospital’s Picture Archiving and Communication System (PACS). To safeguard patient confidentiality, all imaging data were de-identified, with each dataset assigned a unique, anonymous code. Robust security measures were implemented for data storage and access, ensuring the protection and integrity of the information. To standardize the images, noise reduction was applied, and the voxel resolution was resized to 1×1×1 mm³ for uniformity.

### Segmentation and radiomics feature extraction

Tumor regions of interest (ROIs) were delineated using 3D Slicer software (version 4.10.2), with manual segmentation performed on the maximum cross-sectional areas of the CT images. Semi-automated tools, including thresholding and active contour models, were utilized to improve accuracy. The segmentation was carried out by a radiologist (Y.X) with extensive experience in head and neck imaging, who had access to diagnostic information but remained blinded to other clinical details. To evaluate reproducibility, the same radiologist and a skilled surgeon (J.X.) re-segmented the images of 50 randomly selected patients one month later. For radiomics analysis, the Pyradiomics toolkit (version 3.0.1) was used to extract 1,321 features (**Supplementary Table S2**), which included first-order statistics, morphological attributes, texture metrics, and higher-order features generated through advanced image transformations like wavelet and Laplacian of Gaussian filters. These features provided a detailed characterization of tumor heterogeneity, supporting further clinical analysis.

### Evaluation metrics and response assessment

In this study, treatment efficacy was evaluated based on CT imaging features using key metrics: (1) the percentage change in the maximum diameter of the lesion (ΔD%), calculated as (DPre - DPost)/DPre × 100%, and (2) normalized iodine-related attenuation (NIRA), defined as the ratio of iodine concentration in the lesion to that in the common carotid artery, multiplied by 100%. Chemotherapeutic response was classified according to the RECIST criteria into four categories (17): complete response (CR), where all lesions disappeared; partial response (PR), with a reduction in the longest diameter of ≥30%; stable disease (SD), where changes in lesion size did not meet the thresholds for PR or progressive disease (PD); and progressive disease (PD), characterized by an increase in the longest diameter of ≥20% or the emergence of new lesions. CR and PR were considered effective responses, while SD and PD were categorized as ineffective. These metrics provided a structured framework for assessing treatment outcomes and guiding clinical decision-making.

### Feature selection and model development

The radiomics features were refined through a multi-step process to ensure the robustness of the radiomics features, inter- and intra-observer agreement was assessed using the Intraclass Correlation Coefficient (ICC). Only features with an ICC ≥ 0.75 were considered stable and retained for subsequent analysis, while those with an ICC < 0.75 were excluded to minimize variability (**Supplementary Table S3**). To further validate the stability of the selected features and prevent overfitting, we performed bootstrap validation with 1,000 resamples. Further dimensionality reduction was achieved using Spearman’s correlation analysis, Mann-Whitney U tests, and Least Absolute Shrinkage and Selection Operator (LASSO) regression to eliminate redundant variables. Based on the selected features and their corresponding LASSO regression coefficients, a radiomics score (Rad-score) was computed for each patient. The Rad-score was derived using the formula:


$$\text{Rad}-\text{score}=\sum_{i=0}^{n}w_{i}x_{i}^{n}$$

where λi represents the LASSO coefficient of the ith feature and xi denotes its standardized value. To establish an optimal threshold for distinguishing between effective and ineffective chemotherapy responses, receiver operating characteristic (ROC) curve analysis was conducted. The best cutoff value was determined by maximizing the Youden index (sensitivity + specificity − 1), enabling the classification of patients into responder and non-responder groups. This systematic approach ensured the development of a reliable and predictive model for treatment outcome assessment.

### Model construction and validation

To develop predictive models, patients were randomly divided into training and validation sets at a 7:3 ratio using simple random allocation without stratification. Five machine learning algorithms were employed for analysis, including gradient boosting machine (GBM), light gradient boosting machine (LightGBM), extreme gradient boosting (XGBoost), categorical boosting (CatBoost), and random forest (RandomForest). Models were constructed using the Rad-score and other clinical features on the training set, followed by performance evaluation on the validation set. Predictive accuracy was assessed using metrics such as accuracy, sensitivity, specificity, and the area under the receiver operating characteristic curve (AUC). This comprehensive approach ensured the robustness and generalizability of the models in assessing treatment outcomes.

### Tissue collection

All patients underwent surgical procedures where freshly resected tumor tissues were collected post-anesthesia using standardized surgical techniques. The specimens were placed in sterile tubes and rinsed with phosphate-buffered saline (PBS) to remove surface contaminants. Samples were then preserved in RNAlater (Sigma-Aldrich) within sterile empty tubes. To optimize sample integrity, our processing protocol included brief PBS rinses to eliminate intraoperative debris, precise tissue segmentation into small fragments (<5 mm), and flash-freezing in liquid nitrogen vapor, followed by long-term storage at -80 °C. These methodological enhancements were designed to maximize analyte stability for subsequent multi-omics analyses while minimizing pre-analytical variability.

### Transcriptome sequencing and bioinformatics pipeline

Tumor samples for transcriptomic analysis were obtained from pre-treatment biopsy specimens collected prior to the initiation of induction chemotherapy, ensuring that gene expression profiles were not influenced by therapeutic interventions. A total of 23 patients were included in the transcriptomic cohort based on the availability of qualified biopsy specimens. According to the radiomics model, patients were stratified into high and low Rad-score groups, including 10 patients in the high Rad-score group and 13 patients in the low Rad-score group.

RNA samples meeting stringent quality criteria (RIN >7.0, minimum 50 ng/μL concentration) were subjected to poly(A)+ mRNA isolation using oligo-dT magnetic beads. The RNA was then thermally fragmented at 94 °C for 5–7 minutes to prepare strand-specific sequencing libraries. A dUTP-based second-strand synthesis method was employed to preserve strand orientation during downstream analysis. Libraries were sequenced on the Illumina NovaSeq 6000 platform, generating 2×150 bp paired-end reads. Raw sequencing data underwent initial preprocessing with Cutadapt (v3.4) for adapter removal and quality control. HISAT2 (v2.2.1) was used for splice-aware alignment to the GRCh38 human reference genome, followed by transcript-level assembly and quantification using StringTie (v2.1.7) with standard parameters. Differential expression analysis focused on protein-coding transcripts, utilizing FPKM-normalized expression values and applying the Benjamini-Hochberg procedure to control false discovery rates (FDR <0.05).

### Statistical analysis

Statistical analyses were performed using R software (version 4.2.2) and GraphPad Prism (version 9.0). The normality of continuous variables was assessed using the Shapiro-Wilk test and quantile-quantile (Q-Q) plots. Normally distributed data were expressed as mean ± standard deviation (mean ± SD), and parametric analyses, including independent t-tests for two-group comparisons and one-way ANOVA for multi-group comparisons, were conducted. Non-normally distributed data, expressed as median with interquartile range [M (Q1, Q3)], were analyzed using non-parametric methods, including the Mann-Whitney U test for two-group comparisons and the Dunn test for *post hoc* analysis. Categorical variables were reported as frequencies and percentages [n (%)], with nominal data analyzed using Pearson’s chi-square test or Fisher’s exact test, and ordinal data analyzed using the Kruskal-Wallis H test.

Clinical and CT imaging data underwent rigorous quality control and standardized preprocessing to ensure data consistency and reliability. Radiomic feature extraction was performed using the Pyradiomics package, yielding 1,321 features encompassing first-order statistics, morphological, texture, and higher-order filtering features. To identify the most predictive features, a multi-step approach was employed: features with an intraclass correlation coefficient (ICC) > 0.75 were retained for stability, followed by further dimensionality reduction using the Mann-Whitney U test and least absolute shrinkage and selection operator (LASSO) regression. The optimal regularization parameter (α = 0.0652) for LASSO regression was determined through 10-fold cross-validation, ultimately identifying five key features. These features were used to construct a radiomics score (Rad-score), whose discriminative power was validated using the Mann-Whitney U test, revealing significantly higher Rad-scores in the poor prognosis group compared to the favorable prognosis group (*P* < 0.05). Univariate and multivariate logistic regression analyses were conducted to identify independent predictors of chemotherapy response. Five machine learning models (Random Forest, XGBoost, LightGBM, Gradient Boosting Machine, and CatBoost) were developed and compared. Model performance was evaluated using accuracy, sensitivity, specificity, and the area under the receiver operating characteristic curve (AUC). Additionally, SHAP analysis provided interpretability by highlighting the core predictive value of Rad-score, Gap invasion and Cartilage invasion, as well as their synergistic effects. Visualization tools, including bar plots, swarm plots, dependence plots, and waterfall plots, illustrated the individual and combined contributions of these features to the model’s predictions.

## Results

### Study workflow and data characteristics analysis results

**Figure 1:**
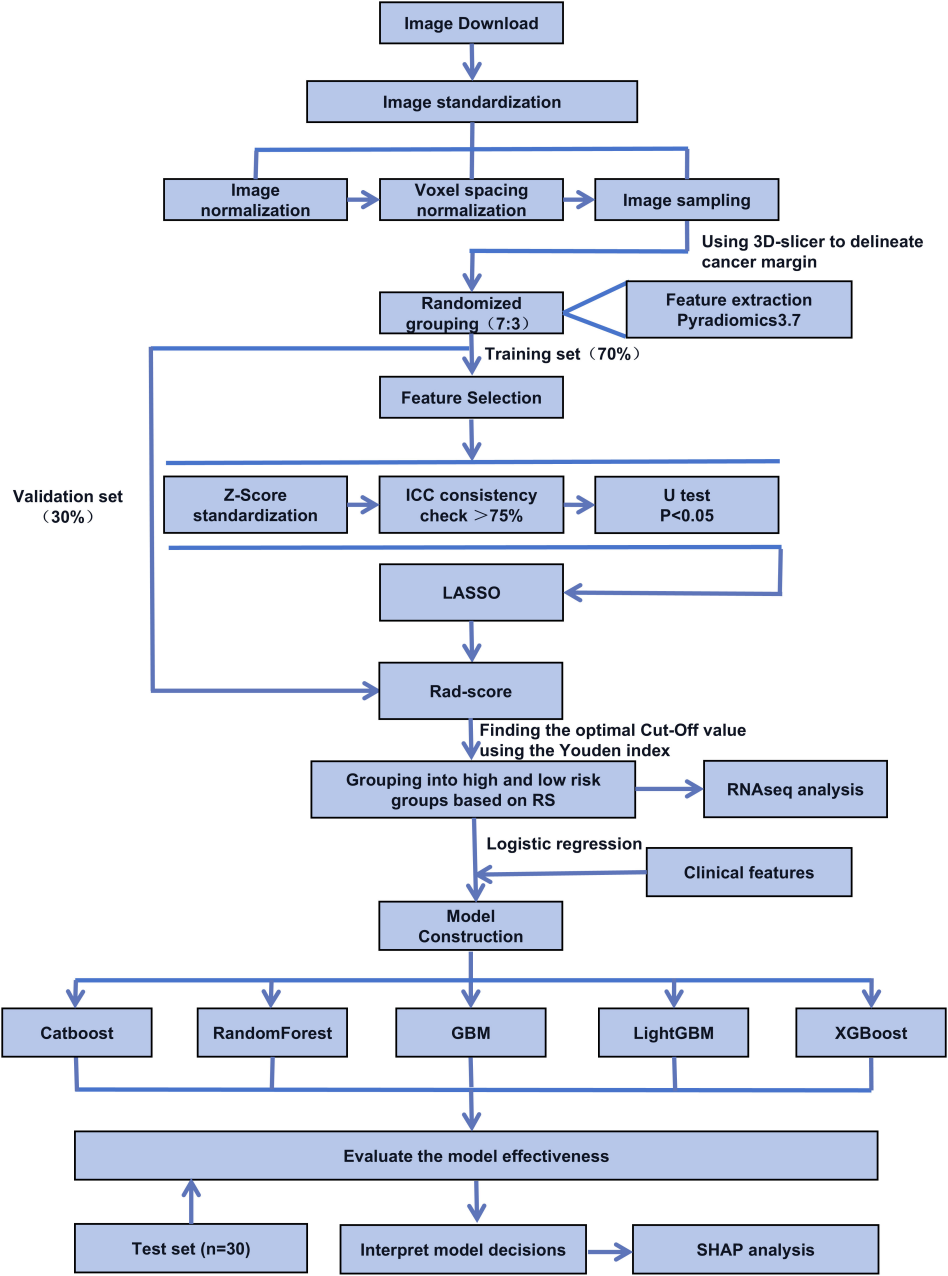
Technical workflow for radiomics model construction and evaluation.

**Figure 1** outlines the study workflow, from medical image standardization and preprocessing to model development and evaluation. Radiomic features were extracted using Pyradiomics, filtered via ICC testing, Mann-Whitney U test, and LASSO regression, and used to construct the radiomics score (Rad-score) for transcriptomic analysis. Clinical data were integrated to build logistic regression, random forest, and XGBoost models, with performance assessed using AUC. **Table 1** shows the baseline clinicopathological characteristics, with no significant differences between training and validation sets in age, gender, tumor type, T/N staging, or Gap invasion status (all P > 0.05), confirming data comparability. Due to the limited sample size, Cartilage invasion was unevenly distributed between the two sets (P <0.001). Among the 131 patients included in this study, 22 patients achieved a complete response (CR), 49 patients achieved a partial response (PR), 43 patients had stable disease (SD), and 17 patients had progressive disease (PD). **Table 2** summarizes the baseline clinical-pathological characteristics of patients stratified by treatment response. Statistical analysis showed that treatment response was significantly associated with T stage, gap invasion, and cartilage invasion (all *P* < 0.05), whereas no significant association was observed for other parameters such as age, sex, N stage, or tumor type.

**Table 1:** Demographics and clinical characteristics of the patients in training and validation set.

Variable	Patients (n=131)	Training set (n=91)	Test set (n=40)	*P* value
Age*/years	62.4 ± 7.7	61.6 ± 7.7	64.1 ± 7.6	0.803
Gender/n, %				
Male	124 (94.6)	84 (92.3)	40 (100.0)	0.071
Female	7 (5.4)	7 (7.7)	0 (0.0)	
Tumor typing/n, %				0.378
Supraglottic type	51 (38.9)	39 (42.9)	12 (30.0)	
Glottic type	71 (54.2)	46 (50.5)	25 (62.5)	
Subglottic type	9 (6.9)	6 (6.6)	3 (7.5)	
T status/n, %				0.381
T1	4 (3.1)	4 (4.4)	0 (0.0)	
T2	29 (22.1)	22 (24.2)	7 (17.5)	
T3	60 (45.8)	41 (45.1)	19 (47.5)	
T4	38 (29.0)	24 (26.4)	14 (35.0)	
N status/n, %				0.533
N0	76 (58.0)	53 (58.2)	23 (57.5)	
N1	31 (23.7)	20 (22.0)	11 (27.5)	
N2	17 (13.0)	14 (15.4)	3 (7.5)	
N3	7 (5.3)	4 (4.4)	3 (7.5)	
Gap invasion				
No	98 (74.8)	25 (27.5)	8 (20.0)	0.364
Yes	33 (25.2)	66 (72.5)	32 (80.0)	
Cartilage invasion				<0.001
No	80 (61.1)	65 (71.4)	15 (37.5)	
Yes	51 (38.9)	26 (28.6)	25 (62.5)	

*age are presented as mean ± SD.

**Table 2:** Comparison of clinical characteristics between patients with well and poor responses to chemotherapy.

Variable	Well response		Poor response		*P* value
	CR	PR	SD	PD	
Age*/years	63.4 ± 7.8	63.0 ± 6.9	61.4 ± 8.0	61.7 ± 9.0	0.299
Gender/n, %					0.162
Male	22 (100.0)	47 (95.9)	40 (93.0)	15 (88.2)	
Female	0 (0.0)	2 (4.1)	3 (7.0)	2 (11.8)	
Tumor typing/n, %					
Supraglottic type	6 (27.3)	18 (36.7)	19 (44.2)	8 (47.1)	0.113
Glottic type	14 (63.6)	30 (61.2)	19 (44.2)	8 (47.1)	
Subglottic type	2 (9.1)	1 (2.0)	5 (11.6)	1 (5.9)	
T status/n, %					0.002
T1	0 (0.0)	4 (8.2)	0 (0.0)	0 (0.0)	
T2	8 (36.4)	14 (28.6)	7 (16.3)	0 (0.0)	
T3	12 (54.5)	20 (40.8)	21 (48.8)	7 (41.2)	
T4	2 (9.1)	11 (22.4)	15 (34.9)	10 (58.8)	
N status/n, %					0.193
N0	19 (86.4)	27 (55.1)	25 (58.1)	5 (29.4)	
N1	1 (4.5)	11 (22.4)	13 (30.2)	6 (35.3)	
N2	2 (9.1)	8 (16.3)	2 (4.7)	5 (29.4)	
N3	0 (0.0)	3 (6.1)	3 (7.0)	1 (5.9)	
Gap invasion					<0.001
No	10 (45.5)	19 (38.8)	3 (7.0)	1 (5.9)	
Yes	12 (54.5)	30 (61.2)	40 (93.0)	16 (94.1)	
Cartilage invasion					<0.001
No	19 (86.4)	35 (71.4)	21 (48.8)	5 (29.4)	
Yes	3 (13.6)	14 (28.6)	22 (51.2)	12 (70.6)	

CR, Complete response; PR, Partial response; SD, Stable disease; PD, Progressive disease.

*age are presented as mean ± SD.

### Key radiomic feature selection and Rad-score construction using LASSO regression

**Figure 2:**
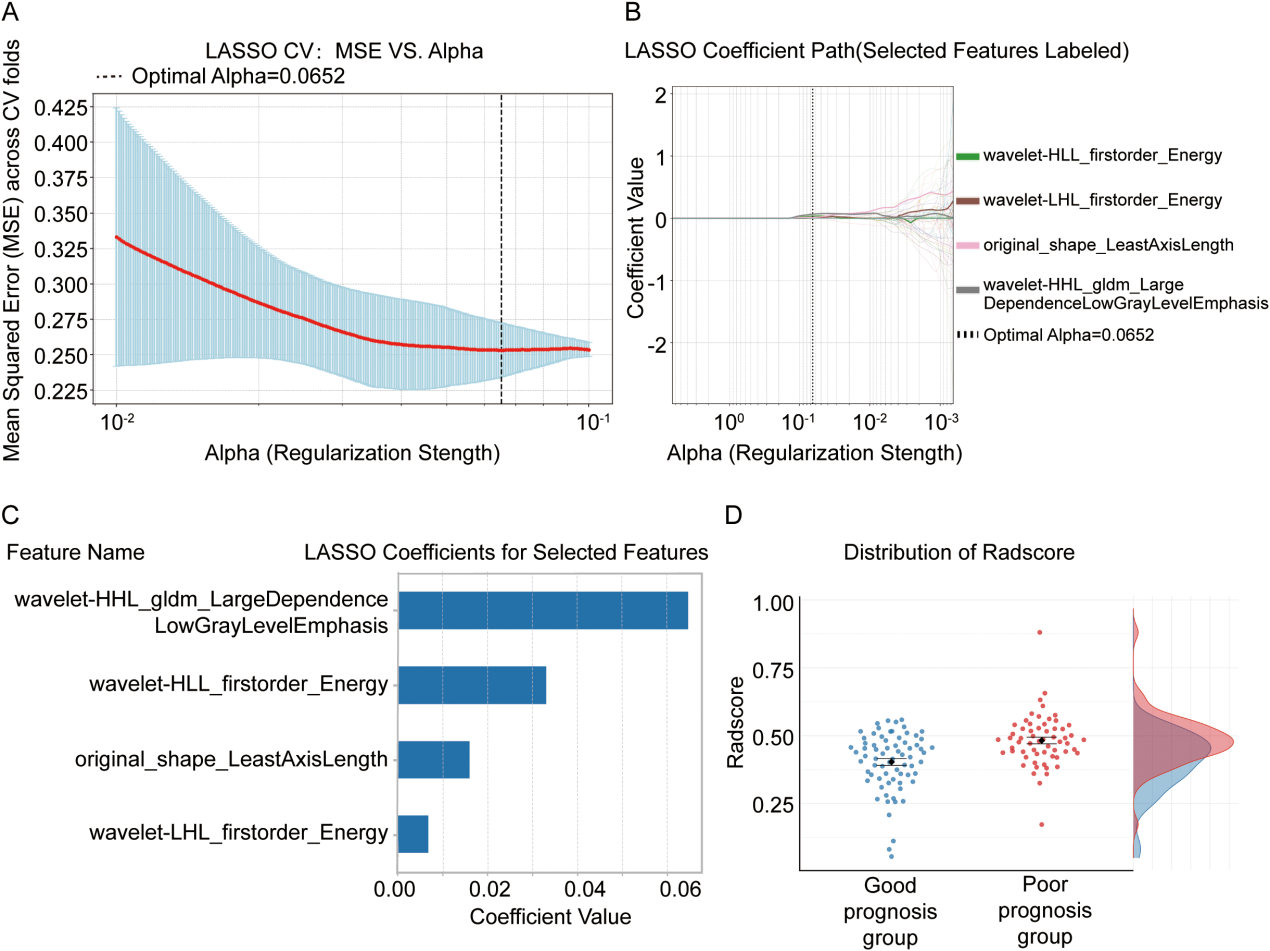
Screening of radiomics features and construction of Rad-score using LASSO regression. **(A, B)** The optimal regularization parameter (α=0.0652) was determined through ten-fold cross-validation (CV), corresponding to the minimum mean squared error (MSE). **(C)** At the optimal α value, the LASSO model selected four radiomics features with non-zero coefficients. **(D)** The radiomics score (Rad-score) constructed based on these five features effectively distinguished the good prognosis group (blue) from the poor prognosis group (red), with the latter exhibiting significantly higher Rad-scores. LASSO, Least Absolute Shrinkage and Selection Operator; CV, cross-validation; MSE, mean squared error; Rad-score, radiomics score.

To identify the most predictive radiomic features for chemotherapy efficacy, we employed a LASSO regression model. Through ten-fold cross-validation, the optimal regularization parameter (α = 0.0652) was determined, corresponding to the minimum mean squared error (**Figure 2A**). At this α value, the LASSO model compressed most feature coefficients to zero, ultimately selecting four radiomic features with non-zero coefficients (**Figure 2B**). These key features and their corresponding LASSO coefficients (**Figure 2C**) included wavelet-HLL gldm Large Dependence Low Gray Level Emphasis, wavelet-HLL firstorder Energy, original shape Least Axis Length, and wavelet-LHL first order Energy (**Figure 2C**). Using these features and their weights, a comprehensive Rad-score was calculated for each patient. The distribution of Rad-score (**Figure 2D**) demonstrated its ability to distinguish between prognosis groups, with significantly higher scores in the poor-prognosis group compared to the good-prognosis group, highlighting its effectiveness in stratifying patients.

### Validation and diagnostic threshold determination of Rad-score for chemotherapy efficacy prediction

**Figure 3:**
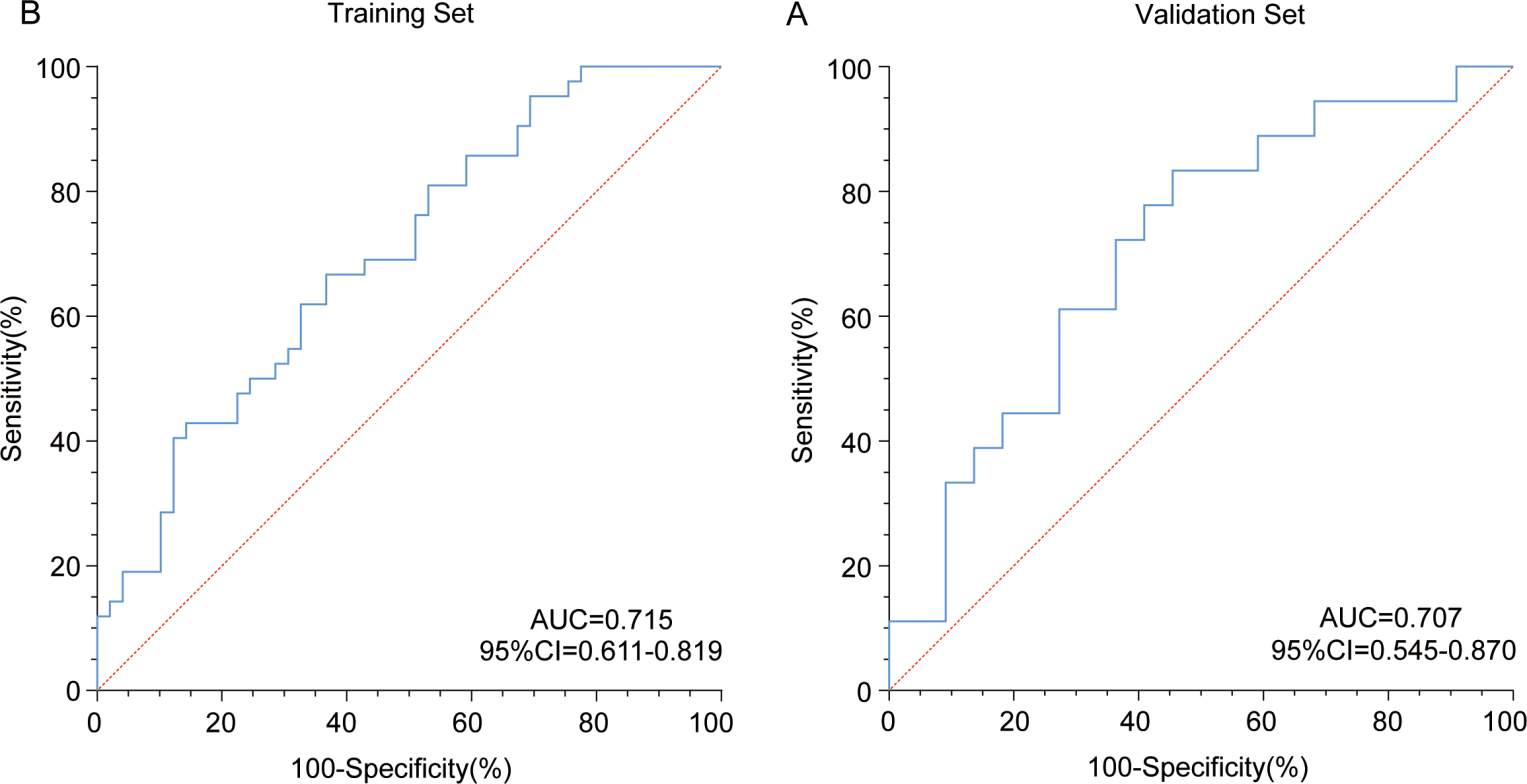
Diagnostic efficacy evaluation of Rad-score. The Rad-score demonstrated diagnostic performance in **(A)** the training set (AUC = 0.715) and **(B)** the validation set (AUC = 0.707) using receiver operating characteristic (ROC) curve analysis.

The Rad-score demonstrated significant potential in predicting chemotherapy efficacy for laryngeal cancer patients. To establish the optimal diagnostic cut-off value, the Youden Index was utilized, revealing a threshold of >0.468, which effectively distinguished treatment outcomes with the highest Youden Index (0.32, **Supplementary Table 4**). Receiver operating characteristic (ROC) curve analysis in the training set showed an area under the curve (AUC) of 0.715 for Rad-score (**Figure 3A**). In the validation set, the AUC was slightly lower at 0.707 (**Figure 3B**), a minor decline typical of machine learning models, indicating robust generalizability and predictive capability. These results confirm the model’s ability to stratify patients based on chemotherapy response. The identified threshold enables objective classification of patients into high-risk (-predicted poor response) and low-risk (predicted favorable response) groups.

### Biological differences and mechanisms revealed by radiomics score group analysis

**Figure 4:**
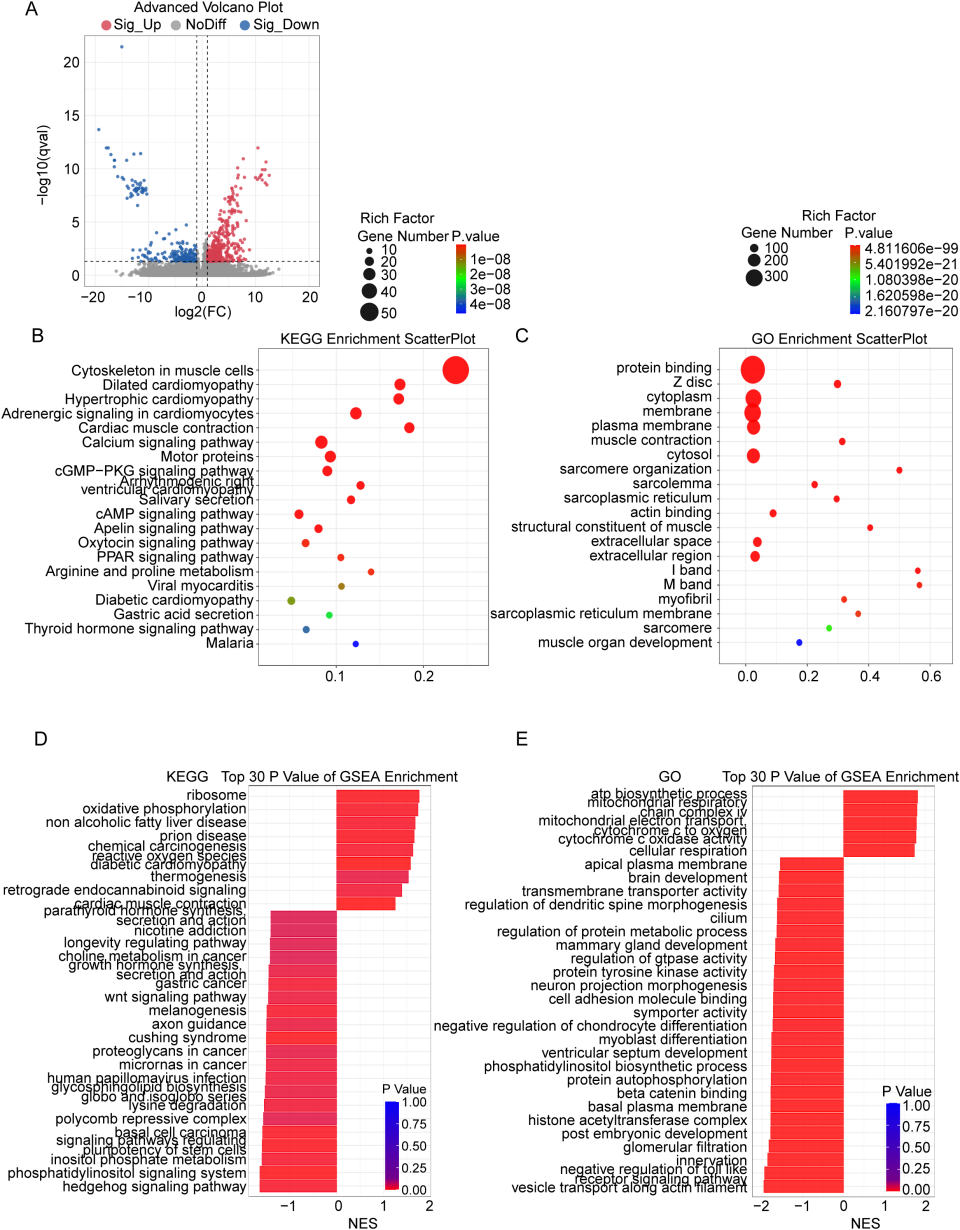
Transcriptomic analysis reveals the biological mechanisms underlying the Rad-score. **(A)** Volcano plot showing DEGs between the high and low Rad-score groups. Upregulated genes in the high Rad-score group are shown in red, while downregulated genes are in blue. **(B)** Bubble plot of KEGG pathway enrichment for the identified DEGs. The bubble size represents the gene count, and the color represents the P-value. **(C)** Bubble plot of GO term enrichment for the identified DEGs, displaying the top biological processes, cellular components, and molecular functions associated with the DEGs. The bubble size corresponds to the gene count, and the color reflects the P-value. **(D)** GSEA of the top 30 enriched KEGG pathways. Pathways enriched in the high Rad-score group (positive NES). The bar color corresponds to the P-value. **(E)** GSEA of the top 30 enriched GO terms. Pathways with a positive NES are enriched in the high Rad-score group, while those with a negative NES are enriched in the low Rad-score group. The bar color indicates the P-value. DEGs, differentially expressed genes; GSEA, Gene Set Enrichment Analysis; GO, Gene Ontology; KEGG, Kyoto Encyclopedia of Genes and Genomes; NES, Normalized Enrichment Score.

Through differential gene expression analysis, pathway enrichment analysis, and tumor microenvironment infiltration analysis of the high and low RS groups, we uncovered significant biological differences and potential underlying mechanisms. Differential gene expression analysis identified numerous significantly differentially expressed genes (DEGs) between the high and low RS groups, indicating that RS score differences are closely associated with extensive transcriptional reprogramming (**Figure 4A**). KEGG pathway enrichment analysis revealed that these DEGs were significantly enriched in pathways related to dilated cardiomyopathy, cardiac muscle contraction, and other muscle functions (**Figures 4B, C**), suggesting that the biological processes in the high RS group may involve abnormalities in cellular structure, energy metabolism, and contractile function. Gene Set Enrichment Analysis (GSEA) further demonstrated that pathways significantly active in the high RS group were primarily associated with metabolism and disease states, such as oxidative phosphorylation and ribosome pathways, while the low RS group was enriched in pathways related to development and signal transduction (**Figures 4D, E**).

### Development and validation of a machine learning model for predicting laryngeal cancer chemotherapy efficacy

To develop a precise clinical decision tool for predicting chemotherapy efficacy in laryngeal cancer patients, this study first identified key independent predictors through univariate and multivariate logistic regression analysis of multiple clinicopathological and imaging features. In the univariate analysis, T status, Gap invasion, Cartilage invasion, and Rad-score were significantly associated with chemotherapy outcome. Specifically, based on the optimal cutoff value determined by the Youden index, patients were stratified into high-risk (Rad-score > 0.468) and low-risk groups. In the multivariate logistic regression analysis (**Table 3**), after adjusting for clinical confounders, the Rad-score > 0.468 remained an independent risk factor (OR = 2.89, 95% CI: 1.29–6.48, *P* = 0.010), alongside Gap invasion (OR = 5.85, *P* = 0.013) and Cartilage invasion (OR = 2.86, *P* = 0.025).

**Table 3:** Logistic regression analyses reveal independent risk predictors associated with chemotherapy efficacy in laryngeal cancer patients.

Variable	Univariate OR (95% CI)	*P*-value	Multivariate OR (95% CI)	*P*-value
Age	0.97 (0.93-1.12)	0.212		
Gender (Male)	3.14 (0.59-16.79)	0.182		
Tumor typing				
Supraglottic type	Ref.	Ref.		
Glottic type	0.55 (0.26-1.13)	0.103		
Subglottic type	1.78 (0.40-7.90)	0.449		
T status	2.60 (1.56-4.31)	<0.001	0.96 (0.46-2.00)	0.911
N status	1.24 (0.84-1.82)	0.275		
Gap invasion	9.67 (3.16-29.61)	<0.001	5.85 (1.46-23.46)	0.013
Cartilage invasion	4.15 (1.97-8.77)	<0.001	2.86 (1.14-7.15)	0.025
Radscore > 0.468	3.57 (1.72-7.40)	0.001	2.89 (1.29-6.48)	0.010

**Figure 5:**
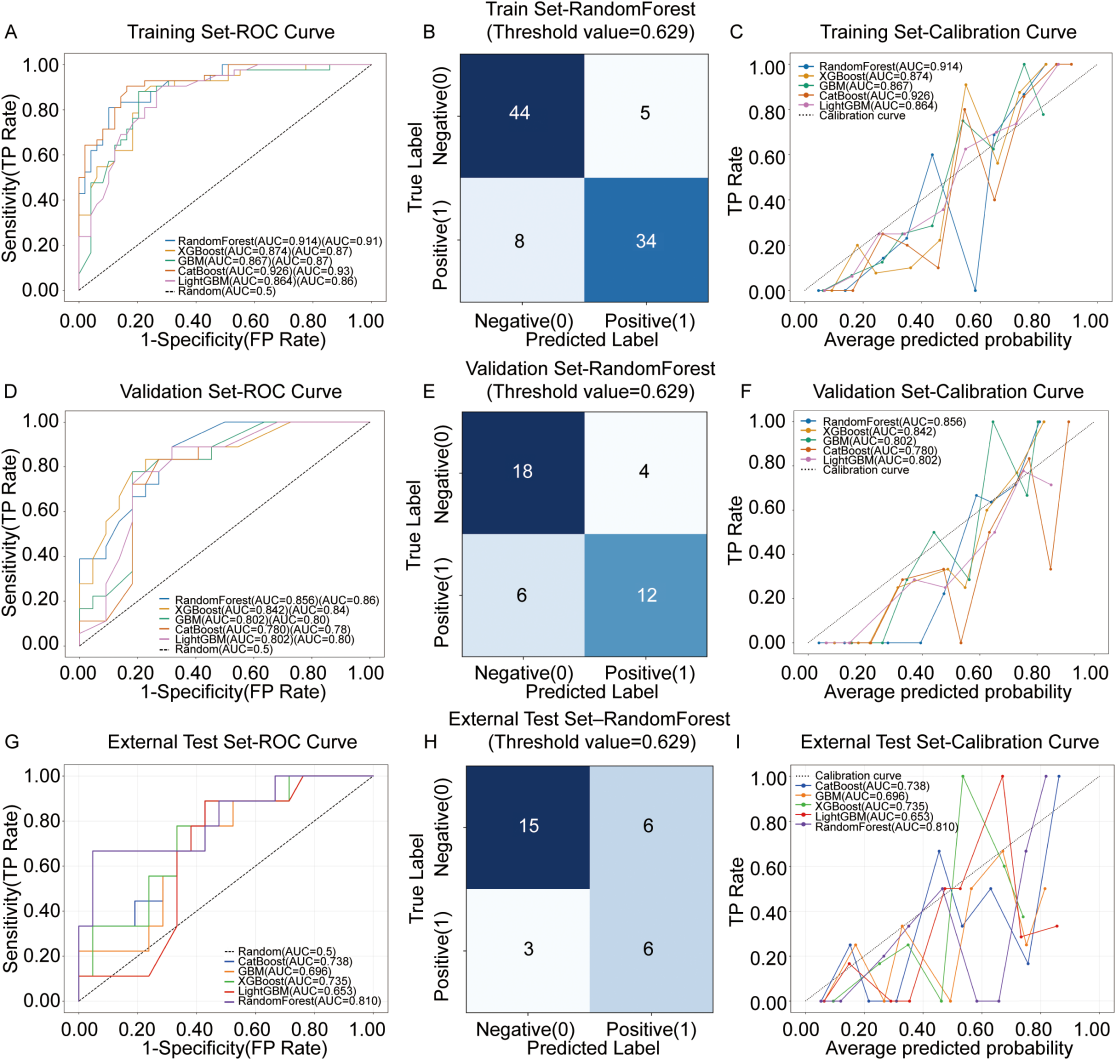
Multi-cohort performance evaluation of machine learning predictive models. **(A, D, G)** Receiver operating characteristic (ROC) curves comparing five machine learning algorithms (RandomForest, XGBoost, GBM, CatBoost, and LightGBM) across the Training **(A)**, Validation **(D)**, and External Test **(G)** sets. While CatBoost showed high performance in the training phase, the RandomForest model (blue curve) demonstrated superior generalization capabilities, achieving robust AUC values of 0.914, 0.856, and 0.810 in the training, validation, and external test cohorts, respectively. **(B, E, H)** Confusion matrices corresponding to the optimal RandomForest model in the Training **(B)**, Validation **(E)**, and External Test **(H)** sets. These matrices illustrate the classification accuracy using an optimized probability threshold of 0.629. **(C, F, I)** Calibration curves assessing the concordance between the predicted probabilities and actual outcomes in the Training **(C)**, Validation **(F)**, and External Test **(I)** sets. The plots indicate that the RandomForest model maintains favorable calibration consistency across different cohorts. ROC, receiver operating characteristic; AUC, area under the curve; GBM, gradient boosting machine.

Subsequently, the study used these identified independent predictors as input features to construct and compare five mainstream machine learning models: Random Forest, XGBoost, LightGBM, Gradient Boosting Machine (GBM), and CatBoost. Evaluated on both the training set and the validation set, the Random Forest model demonstrated the best overall performance (**Figures 5A, D**; **Supplementary Table S5**). In the training set, the Random Forest model achieved an impressive ROC AUC of 0.91. In the validation set, it maintained a robust AUC of 0.86, significantly outperforming other models and demonstrating strong generalizability and reliability. At the optimal threshold (0.629), the Random Forest model achieved an accuracy of 85.7%, a sensitivity of 80.9%, and a specificity of 89.7% on the test set. The confusion matrix clearly illustrated its high effectiveness in correctly identifying patients with poor treatment response (12 true positives) and favorable response (18 true negatives) (**Figures 5B, E**; **Supplementary Table S6**).

Calibration curves further confirmed the strong alignment between the model’s predicted probabilities and actual observations, highlighting its reliability (**Figures 5C, F**). To further verify the model’s robustness, we applied the trained Random Forest model to the independent external test set (n=30). As shown in **Figure 5G**, the model achieved a promising AUC of 0.810. The confusion matrix (**Figure 5H**) indicated a high correct classification rate with 15 True Negatives and 6 True Positives. The calibration curve (**Figure 5I**) in the external set also showed good agreement between predicted probabilities and observed outcomes. This suggests that our radiomics-based model maintains high predictive value even in unseen data.

Stratified analysis was performed to evaluate the association between model-derived risk groups and clinicopathological variables. Patients classified into the high-risk group were significantly associated with advanced T stage and higher N stage (both P<0.001, chi-square test), indicating a greater locoregional disease burden. In contrast, no significant differences were observed between risk groups with respect to age (*P* = 0.077, t-test), gender (*P* = 0.708), or primary tumor subsite (*P* = 0.369). These findings suggest that the radiomics model–predicted high-risk category is correlated with more advanced locoregional disease extent, while remaining independent of demographic characteristics and primary tumor subsite (**Table 4**).

**Table 4:** Comparison of clinical characteristics between different risk groups defined by the radiomics model.

Variable	Low risk (n=50)	High risk (n=81)	*P* value
Age*/years	62.8 ± 6.8	62.2 ± 8.2	0.077
Gender/n, %			
Male	48 (96.0)	76 (93.8)	0.708
Female	2 (4.0)	5 (6.2)	
Tumor typing/n, %			
Supraglottic type	16 (32.0)	35 (43.2)	0.369
Glottic type	31 (62.0)	40 (49.4)	
Subglottic type	3 (6.0)	6 (7.4)	
T status/n, %			
T1	4 (8.0)	0 (0.0)	<0.001
T2	23 (46.0)	6 (7.4)	
T3	20 (40.0)	40 (49.4)	
T4	3 (6.0)	35 (43.2)	
N status/n, %			
N0	40 (80.0)	36 (44.4)	<0.001
N1	4 (8.0)	27 (33.3)	
N2	5 (10.0)	12 (14.8)	
N3	1 (2.0)	6 (7.4)	

*age are presented as mean ± SD.

### SHAP analysis of a machine learning model for laryngeal cancer

**Figure 6:**
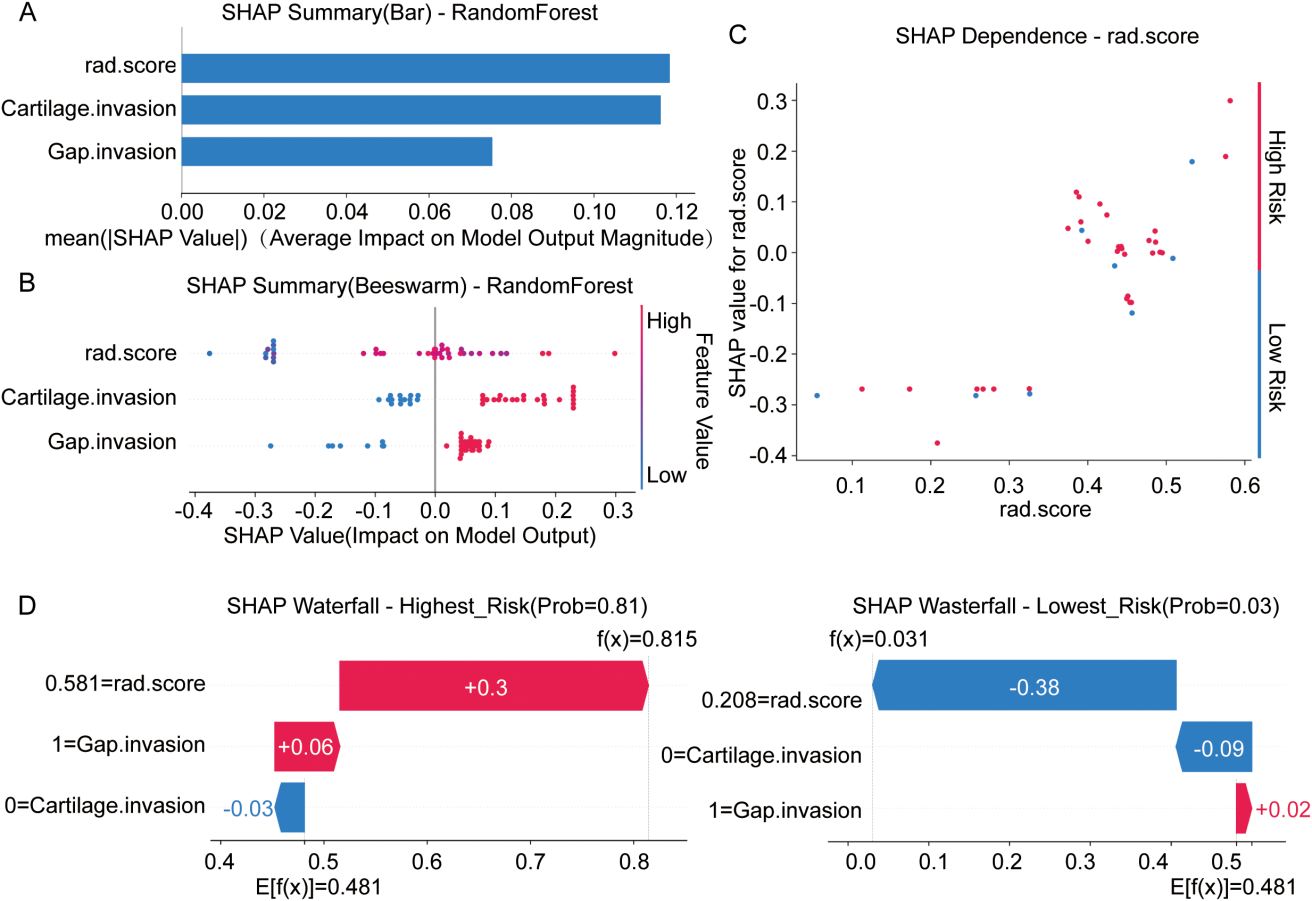
SHAP analysis for interpreting the optimal RandomForest model. **(A)** SHAP summary bar plot ranking the features based on their average absolute impact on the model output. rad.score and Cartilage.invasion are identified as the most influential predictors. **(B)** SHAP beeswarm plot illustrating the distribution of feature impacts. Each dot represents a sample; red indicates a high feature value (e.g., presence of invasion or high rad-score), which correlates with a positive SHAP value (higher risk). **(C)** SHAP dependence plot for rad.score, showing how the risk contribution (y-axis) changes as the feature value (x-axis) increases. **(D)** SHAP waterfall plots for two representative individual cases. Left: A high-risk patient (Probability = 0.81) where a high rad.score (+0.3 contribution) pushes the prediction higher. Right: A low-risk patient (Probability = 0.03) where a low rad.score (-0.38 contribution) drives the risk down. The base value (E [ f(x)]=0.481) represents the average model prediction across the dataset.

To deeply understand the internal decision-making mechanism of the optimal RandomForest model, this study employed SHAP (SHapley Additive exPlanations) analysis to quantify the contribution and direction of each feature to the prediction outcomes. The SHAP summary bar plot (**Figure 6A**) identified rad.score and Cartilage.invasion as the two dominant predictors in the model, followed by Gap.invasion. The beeswarm plot (**Figure 6B**) further visualized the directional impact of these features: higher rad.score values and the presence of invasion (indicated by red dots for Cartilage.invasion and Gap.invasion) were distributed on the positive side of the x-axis, signifying a substantial contribution to an increased risk of poor chemotherapy response. Additionally, the SHAP dependence plot (**Figure 6C**) revealed a non-linear relationship for the rad.score: once the score exceeds a threshold of approximately 0.35, the risk contribution rises sharply, indicating a distinct tipping point for malignancy.

To demonstrate the model’s transparency at the individual level, we utilized SHAP waterfall plots (**Figure 6D**) to visualize decision paths for specific patients. In a representative high-risk case (Left panel), the model predicted a probability of 0.815 (far exceeding the base value of 0.481). This elevated risk was primarily driven by a high rad.score (0.581), which contributed +0.3 to the risk score. Conversely, in a low-risk example (Right panel), a low rad.score (0.208) significantly reduced the predicted risk by -0.38, resulting in a final probability of just 0.031. In conclusion, SHAP analysis not only confirmed the predictive robustness of rad.score and invasive features but also successfully transformed the “black box” RandomForest model into an interpretable tool, clearly delineating the specific reasoning behind each clinical prediction.

## Discussion

This study successfully developed and validated a robust, non-invasive predictive model for chemotherapy response in patients with advanced laryngeal cancer. Our key finding is that a CatBoost machine learning model integrating a novel CT radiomics score (Rad-score) and the clinical feature “ tumor invasion of the fat gap” (Gap invasion) achieved exceptional accuracy in predicting induction chemotherapy efficacy, with an AUC of 0.84 in the validation set (18). More importantly, through transcriptomic analysis, we revealed for the first time the profound biological basis underlying the abstract imaging indicator Rad-score, linking it to an aggressive tumor phenotype characterized by cellular remodeling, metabolic hyperactivity, and signaling pathway reprogramming. These findings mark a critical step toward personalized treatment stratification for this challenging disease.

Previous studies have demonstrated the potential of CT-based radiomics to predict chemotherapy response in laryngeal and head and neck cancers. For example, multicenter studies have shown that statistical harmonization techniques, such as ComBat combined with unsupervised clustering, improve the predictive value of radiomic features in heterogeneous datasets (19). Single-center studies have developed radiomics nomograms integrating imaging features with clinicopathological factors, showing predictive value for pathological response and overall survival (20). More recently, multiomic analyses have demonstrated that combining clinical, genomic, and radiomic data enhances predictive accuracy for chemotherapy response and laryngo-esophageal dysfunction (21). Our findings indicate that more advanced local tumor invasion is associated with a poorer response to induction chemotherapy, and highlight the potential clinical utility of the CT-based Rad-score in predicting chemotherapy response in patients with laryngeal cancer. Notably, the Random Forest model incorporating the Rad-score, gap invasion, and cartilage invasion achieved the best predictive performance in both the training and validation sets, and its predictive efficacy was further confirmed in an independent testing cohort. These findings are also consistent with previous multicenter and single-center radiomics studies.

A distinctive aspect of our study is the integration of transcriptomic profiling to decode the biological underpinnings of the Rad-score. While the radiomics signature (Rad-score) demonstrated robust predictive power in both training and validation sets (AUC = 0.715 and 0.707) (22), its true value lies in decoding the biological underpinnings of these imaging traits. Integrated transcriptomic profiling reveals that a high Rad-score serves as a surrogate for a specific “Metabolic-Structural Resistant Phenotype” (23). Mechanistically, this high-risk state is driven by a dual resistance engine. First, metabolic reprogramming shifts cells toward a hyper-active energy status, evidenced by the significant upregulation of Oxidative Phosphorylation (OXPHOS) and Ribosome pathways (**Figures 4D, E**) (24–26). This high-OXPHOS state meets the immense energy demands for DNA repair and drug efflux, while enhanced ribosome biogenesis facilitates the rapid replacement of proteins damaged by cytotoxic stress (25, 26). Second, profound structural remodeling forms a physical line of defense. Notably, the enrichment of pathways classically categorized as “cardiomyopathy” (e.g., Hypertrophic and Dilated Cardiomyopathy, **Figure 4B**) in our laryngeal cancer cohorts fundamentally reflects the extensive activation of conserved actomyosin structures (27), motor proteins (28), and calcium signaling modules (29). In the malignant context, these components—traditionally associated with muscle contraction—are hijacked by tumor cells to drive the cytoskeletal reorganization required for epithelial-mesenchymal transition (EMT) and to modulate intracellular calcium flux, a key regulator of multidrug resistance (MDR) proteins and anti-apoptotic signaling (28–30). Consequently, the Rad-score identifies tumors that are biologically “combat-ready”—armed with the metabolic reserves and structural plasticity necessary to withstand systemic therapy. Thus, our Rad-score is no longer a black box metric but an effective window for non-invasively assessing the intrinsic aggressive biological behavior of tumors, signifying a tumor state biologically predisposed to treatment failure, providing additional mechanistic insights through the integration of transcriptomic data.

Multivariate logistic regression analysis confirmed that the Rad-score, gap invasion, and cartilage invasion are robust independent predictors of chemotherapy response in advanced laryngeal cancer. Specifically, the Rad-score demonstrated a significant independent predictive value with an Odds Ratio (OR) of 2.89 (95% CI: 1.29–6.48, P = 0.010). This effect size, achieved after rigorous quality control—including an elevated ICC threshold of 0.75 and bootstrap validation—reflects a stable biological correlation with tumor heterogeneity while effectively mitigating the risk of overfitting common in radiomics studies. Clinical indicators of local spread, namely gap invasion (OR = 5.85) and cartilage invasion (OR = 2.86), further complemented the imaging metrics, aligning with clinical observations that anatomically aggressive tumors present greater therapeutic challenges (31–33).

Building upon these robust predictors, the Random Forest model emerged as the superior performer among the five tested algorithms, achieving an impressive AUC of 0.856 in the validation set and maintaining a steady AUC of 0.810 in the independent external test set. Notably, the model’s exceptionally high specificity (89.7%) is clinically pivotal; it ensures the reliable identification of true responders, thereby providing strong evidence to support larynx-preserving strategies and preventing the overtreatment of patients who would not benefit from induction chemotherapy. To bridge the gap between complex machine learning and clinical practice, we employed SHAP analysis to deconstruct this “black box” (34, 35). By transparently quantifying the contributions of the Rad-score and invasive features, SHAP revealed a significant synergistic effect and a distinct “risk tipping point” at a Rad-score of 0.35. This interpretability not only enhances the model’s credibility but also provides a practical, non-invasive tool for precision oncology, empowering clinicians to make data-driven, personalized treatment decisions (36).

Although an independent external test set was included in this study, all patients involved in our study were derived from a single center, which may limit the generalizability of the model. In addition, the sample size of the external validation cohort was relatively small. While the model achieved an AUC of 0.810 in the external dataset, indicating relatively stable predictive performance, these findings should be interpreted with caution. Further validation in larger, multicenter cohorts is necessary to confirm the robustness and generalizability of the proposed radiomics model.

## Conclusions

In this study, we successfully developed a highly accurate and interpretable Random Forest model to predict the response to induction chemotherapy in patients with advanced laryngeal cancer. The strength of this model lies in its integration of a radiomics signature with clear biological significance—capturing features of tumor metabolism, structural remodeling, and signaling pathway reprogramming—and a key clinical marker of tumor aggressiveness. This non-invasive tool enables precise risk stratification before treatment begins, empowering clinicians to tailor more individualized therapeutic strategies. As a result, it has the potential to spare predicted non-responders from the toxic side effects of ineffective chemotherapy and guide them toward more appropriate interventions promptly. Future prospective studies are essential to ultimately confirm the practical value of this model in improving patient clinical outcomes.

## Data Availability

The data generated and/or analysed during the current study are available in the China National Center for Bioinformation (HRA013938).

## Supplementary material

Supplementary Table S1 Demographics and clinical feature of the study.

Supplementary Table S2 Radiomics characteristics of the training and validation set.

Supplementary Table S3 ICC consistency verification.

Supplementary Table S4 Yoden index of RS in training set.

Supplementary Table S5 Radiomics characteristics of the test set.

Supplementary Table S6 Comparison of machine learning efficiency.

## References

[1] Siegel RL Miller KD Wagle NS Jemal A . Cancer statistics, 2023. CA Cancer J Clin. (2023) 73:17–48. doi: 10.3322/caac.21763, PMID: 36633525

[2] Mody M Rocco J Yom S Haddad R Saba NJL . Head and neck cancer. (2021) 398:2289–99. doi: 10.1016/s0140-6736(21)01550-6, PMID: 34562395

[3] Forastiere AA Goepfert H Maor M Pajak TF Weber R Morrison W . Concurrent chemotherapy and radiotherapy for organ preservation in advanced laryngeal cancer. N Engl J Med. (2003) 349:2091–8. doi: 10.1056/NEJMoa031317, PMID: 14645636

[4] Lefebvre JL Pointreau Y Rolland F Alfonsi M Baudoux A Sire C . Induction chemotherapy followed by either chemoradiotherapy or bioradiotherapy for larynx preservation: the TREMPLIN randomized phase II study. J Clin Oncol. (2013) 31:853–9. doi: 10.1200/jco.2012.42.3988, PMID: 23341517

[5] Okano S Homma A Kiyota N Tahara M Hanai N Asakage T . Induction chemotherapy in locally advanced squamous cell carcinoma of the head and neck. Jpn J Clin Oncol. (2021) 51:173–9. doi: 10.1093/jjco/hyaa220, PMID: 33290543

[6] Lambin P Leijenaar RTH Deist TM Peerlings J de Jong EEC van Timmeren J . Radiomics: the bridge between medical imaging and personalized medicine. Nat Rev Clin Oncol. (2017) 14:749–62. doi: 10.1038/nrclinonc.2017.141, PMID: 28975929

[7] Jiang Y Zhou K Sun Z Wang H Xie J Zhang T . Non-invasive tumor microenvironment evaluation and treatment response prediction in gastric cancer using deep learning radiomics. Cell Rep Med. (2023) 4:101146. doi: 10.1016/j.xcrm.2023.101146, PMID: 37557177 PMC10439253

[8] Gillies RJ Kinahan PE Hricak H . Radiomics: images are more than pictures, they are data. Radiology. (2016) 278:563–77. doi: 10.1148/radiol.2015151169, PMID: 26579733 PMC4734157

[9] Aerts HJ Velazquez ER Leijenaar RT Parmar C Grossmann P Carvalho S . Decoding tumour phenotype by noninvasive imaging using a quantitative radiomics approach. Nat Commun. (2014) 5:4006. doi: 10.1038/ncomms5006, PMID: 24892406 PMC4059926

[10] O'Connor JP Aboagye EO Adams JE Aerts HJ Barrington SF Beer AJ . Imaging biomarker roadmap for cancer studies. Nat Rev Clin Oncol. (2017) 14:169–86. doi: 10.1038/nrclinonc.2016.162, PMID: 27725679 PMC5378302

[11] Huang SH O'Sullivan B . Overview of the 8th edition TNM classification for head and neck cancer. Curr Treat Options Oncol. (2017) 18:40. doi: 10.1007/s11864-017-0484-y, PMID: 28555375

[12] Inchingolo R Maino C Cannella R Vernuccio F Cortese F Dezio M . Radiomics in colorectal cancer patients. World J Gastroenterol. (2023) 29:2888–904. doi: 10.3748/wjg.v29.i19.2888, PMID: 37274803 PMC10237092

[13] Berz AM Dromain C Vietti-Violi N Boughdad S Duran R . Tumor response assessment on imaging following immunotherapy. Front Oncol. (2022) 12:982983. doi: 10.3389/fonc.2022.982983, PMID: 36387133 PMC9641095

[14] Liu X Xu Y Shu J Zuo Y Li Z Lin M . Preoperative CT and radiomics nomograms for distinguishing bronchiolar adenoma and early-stage lung adenocarcinoma. Acad Radiol. (2025) 32:1054–66. doi: 10.1016/j.acra.2024.08.047, PMID: 39256085

[15] Zhang X Lu B Yang X Lan D Lin S Zhou Z . Prognostic analysis and risk stratification of lung adenocarcinoma undergoing EGFR-TKI therapy with time-serial CT-based radiomics signature. Eur Radiol. (2023) 33:825–35. doi: 10.1007/s00330-022-09123-5, PMID: 36166088 PMC9889474

[16] Tang LQ Chen DP Guo L Mo HY Huang Y Guo SS . Concurrent chemoradiotherapy with nedaplatin versus cisplatin in stage II-IVB nasopharyngeal carcinoma: an open-label, non-inferiority, randomised phase 3 trial. Lancet Oncol. (2018) 19:461–73. doi: 10.1016/s1470-2045(18)30104-9, PMID: 29501366

[17] Hu Z Chen Y Ma R Sun W Chen L Cai Z . Nomogram prediction of response to neoadjuvant chemotherapy plus pembrolizumab in locally advanced hypopharyngeal squamous cell carcinoma. J Otolaryngol Head Neck Surg. (2025) 54:19160216251318255. doi: 10.1177/19160216251318255, PMID: 39921555 PMC11807280

[18] Abbas GH Khouri ER Thaher O Taha S Vladimirov M Oviedo RJ . Predictive modeling for metastasis in oncology: current methods and future directions. Ann Med Surg (Lond). (2025) 87:3489–508. doi: 10.1097/ms9.0000000000003279, PMID: 40486555 PMC12140723

[19] Masson I Da-Ano R Lucia F Doré M Castelli J Goislard de Monsabert C . Statistical harmonization can improve the development of a multicenter CT-based radiomic model predictive of nonresponse to induction chemotherapy in laryngeal cancers. Med Phys. (2021) 48:4099–109. doi: 10.1002/mp.14948, PMID: 34008178

[20] Kang C Sun P Yang R Zhang C Ning W Liu H . CT radiomics nomogram predicts pathological response after induced chemotherapy and overall survival in patients with advanced laryngeal cancer: A single-center retrospective study. Front Oncol. (2023) 13:1094768. doi: 10.3389/fonc.2023.1094768, PMID: 37064100 PMC10103838

[21] Mattavelli D Compagnoni M Calza S Ravanelli M Plana M Wichmann G . A multiomic framework for predicting laryngo-esophageal dysfunction following induction chemotherapy in hypopharyngeal-laryngeal carcinoma. ESMO Open. (2026) 11:105933. doi: 10.1016/j.esmoop.2025.105933, PMID: 41468687 PMC12804047

[22] Kawahara D Kishi M Kadooka Y Hirose K Murakami Y . Integrating radiomics and gene expression by mapping on the image with improved DeepInsight for clear cell renal cell carcinoma. Cancer Genet. (2025) 292-293:100–5. doi: 10.1016/j.cancergen.2025.02.004, PMID: 39983665

[23] Sarkar FH Li Y Wang Z Kong D . The role of nutraceuticals in the regulation of Wnt and Hedgehog signaling in cancer. Cancer Metastasis Rev. (2010) 29:383–94. doi: 10.1007/s10555-010-9233-4, PMID: 20711635 PMC2974632

[24] Deng M Zhou Z Chen J Li X Liu Z Ye J . Enhanced oxidative phosphorylation driven by TACO1 mitochondrial translocation promotes stemness and cisplatin resistance in bladder cancer. Adv Sci (Weinh). (2025) 12:e2408599. doi: 10.1002/advs.202408599, PMID: 39656941 PMC11791945

[25] Zang Y Ran X Yuan J Wu H Wang Y Li H . Genomic hallmarks and therapeutic targets of ribosome biogenesis in cancer. Brief Bioinform. (2024) 25. doi: 10.1093/bib/bbae023, PMID: 38343327 PMC10859687

[26] Hagen JT Montgomery MM Aruleba RT Chrest BR Krassovskaia P Green TD . Acute myeloid leukemia mitochondria hydrolyze ATP to support oxidative metabolism and resist chemotherapy. Sci Adv. (2025) 11:eadu5511. doi: 10.1126/sciadv.adu5511, PMID: 40203117 PMC11980858

[27] Li Y Wang D Ge H Güngör C Gong X Chen Y . Cytoskeletal and cytoskeleton-associated proteins: key regulators of cancer stem cell properties. Pharm (Basel). (2022) 15. doi: 10.3390/ph15111369, PMID: 36355541 PMC9698833

[28] Guan F Wu X Zhou J Lin Y He Y Fan C . Mitochondrial transfer in tunneling nanotubes-a new target for cancer therapy. J Exp Clin Cancer Res. (2024) 43:147. doi: 10.1186/s13046-024-03069-w, PMID: 38769583 PMC11106947

[29] Lin J Wang X Ma S Yang D Li K Li D . Calcium channels as therapeutic targets in head and neck squamous cell carcinoma: current evidence and clinical trials. Front Oncol. (2024) 14:1516357. doi: 10.3389/fonc.2024.1516357, PMID: 39759147 PMC11695298

[30] Guan R Li C Jiao R Li J Wei R Feng C . MRPL21-PARP1 axis promotes cisplatin resistance in head and neck squamous cell carcinoma by inhibiting autophagy through the PI3K/AKT/mTOR signaling pathway. J Exp Clin Cancer Res. (2025) 44:221. doi: 10.1186/s13046-025-03482-9, PMID: 40713706 PMC12297673

[31] Ferrari M Mularoni F Smussi D Gaudioso P Bonomo P Friborg J . International consensus on laryngeal preservation strategies in laryngeal and hypopharyngeal cancer. Lancet Oncol. (2025) 26:e264–81. doi: 10.1016/s1470-2045(25)00020-8, PMID: 40318658

[32] Wang X Yang Y Wu J Tang X Wang Y . Prediction of metastatic risk of renal clear cell carcinoma based on CT radiomics analysis. Front Oncol. (2025) 15:1576956. doi: 10.3389/fonc.2025.1576956, PMID: 40548117 PMC12178895

[33] Adams SC Nambiar AK Bressler EM Raut CP Colson YL Wong WW . Immunotherapies for locally aggressive cancers. Adv Drug Delivery Rev. (2024) 210:115331. doi: 10.1016/j.addr.2024.115331, PMID: 38729264 PMC11228555

[34] Cui J Liu G Yue K Wu Y Duan Y Wei M . Development and validation of an explainable machine learning model to predict Delphian lymph node metastasis in papillary thyroid cancer: a large cohort study. J Cancer. (2025) 16:2041–61. doi: 10.7150/jca.110141, PMID: 40092685 PMC11905415

[35] Liu Y Fu Y Peng Y Ming J . Clinical decision support tool for breast cancer recurrence prediction using SHAP value in cooperative game theory. Heliyon. (2024) 10:e24876. doi: 10.1016/j.heliyon.2024.e24876, PMID: 38312672 PMC10835316

[36] Chen W Jia Z Wan L Yuan W Xu J Du X . Interpretable radiomics model based on dual-layer spectral CT iodine maps for predicting microsatellite instability in colorectal cancer: A two-center study. Eur J Radiol. (2025) 192:112357. doi: 10.1016/j.ejrad.2025.112357, PMID: 40886491

